# Upper gastrointestinal ischemia as a rare complication of paroxysmal nocturnal hemoglobinuria

**DOI:** 10.1002/ccr3.3567

**Published:** 2020-11-22

**Authors:** Masayuki Ueno, Yuichi Shimodate, Kazuya Okada, Ryosuke Takaya, Hiroshi Yamamoto, Motowo Mizuno

**Affiliations:** ^1^ Department of Gastroenterology and Hepatology Kurashiki Central Hospital Kurashiki Japan; ^2^ Department of Hematology/Oncology Kurashiki Central Hospital Kurashiki Japan

**Keywords:** esophagogastroduodenoscopy, gastrointestinal ischemia, *Helicobacter pylori* gastritis, paroxysmal nocturnal hemoglobinuria, thromboembolism

## Abstract

When patients with PNH present with abdominal symptoms, thrombosis‐induced gastrointestinal injury should be considered; computed tomography and esophagogastroduodenoscopy may help make the diagnosis of this potentially serious complication.

## Graphical abstract

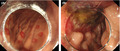

## 1 INTRODUCTION

Here, we report a case of PNH with gastroduodenal lesions likely caused by transient mucosal ischemia due to thrombosis. When patients with PNH present with abdominal symptoms, thrombosis‐induced gastrointestinal injury should be considered. Recognition of gastric patchy redness on esophagogastroduodenoscopy may help make the diagnosis of this potentially serious complication.

Paroxysmal nocturnal hemoglobinuria (PNH) is a clonal hematopoietic stem cell disorder that is characterized by hemolytic anemia, bone marrow failure, and thrombosis.^1^ Thromboembolism in PNH is associated with poor prognosis and is a leading cause of death.^2^, ^3^ Making prompt diagnosis of thrombosis is important to help determine the treatment for patients with PNH.^4^ However, making the diagnosis is sometimes challenging, partly because of caregivers’ unfamiliarity with its clinical manifestations. Despite the high frequency of intraabdominal thrombosis in PNH,^2^ reports of upper gastrointestinal manifestations of thrombotic complications are rare.^5^ Here, we report a patient with PNH who developed gastric and duodenal lesions likely caused by transient mucosal ischemia due to PNH‐related thrombosis. This case report was prepared according to the CARE Guidelines.^6^

## 2 CASE REPORT

A 64‐year‐old woman presented to the emergency department with fever and dark urine of one week's duration. Two years earlier, PNH was diagnosed, based on flow cytometry findings of peripheral blood. Although thrombocytopenia had been present, no specific therapy was necessary. Her past medical history included hyperlipidemia and *Helicobacter pylori*–induced gastritis; *H pylori* had been eradicated. She had a temperature of 38.2°C and tenderness in the left costovertebral angle. Laboratory tests revealed severe anemia, thrombocytopenia, and coagulopathy (Table 1). C‐reactive protein was 33.2 mg/dL [normal range, 0.00‐0.14], lactate dehydrogenase 802 U/L [124‐222], and serum creatinine 3.81 mg/dL [0.46‐0.79]. Blood and urine cultures were negative, probably because she had taken oral antibiotics before presentation. Bacterial infection–triggered hemolysis, possibly from pyelonephritis, was suspected.

**Table 1:** Laboratory data before and after treatment

	Normal range	At admission	3 weeks later
Hb (g/dL)	11.6‐14.8	4.5	7.5
WBC (/μL)	3300‐8600	12 400	4600
PLT (×10^4^/μL)	16.0‐36.0	1.5	4.2
PT‐INR	0.91‐1.14	1.21	1.00
APTT (s)	26.9‐38.1	27.4	<20.0
FDP (μg/mL)	0.0‐5.0	81.3	3.1
D‐dimer (μg/mL)	0.0‐1.0	35.0	1.0
Haptoglobin (mg/dL)	19.0‐170.0	<1.0	2.0

Abbreviations: APTT, activated partial thromboplastin time; FDP, fibrinogen degradation products; Hb, hemoglobin; PLT, platelet count; PT‐INR, prothrombin time‐international normalized ratio; WBC, white blood cell.

**Figure 1:**
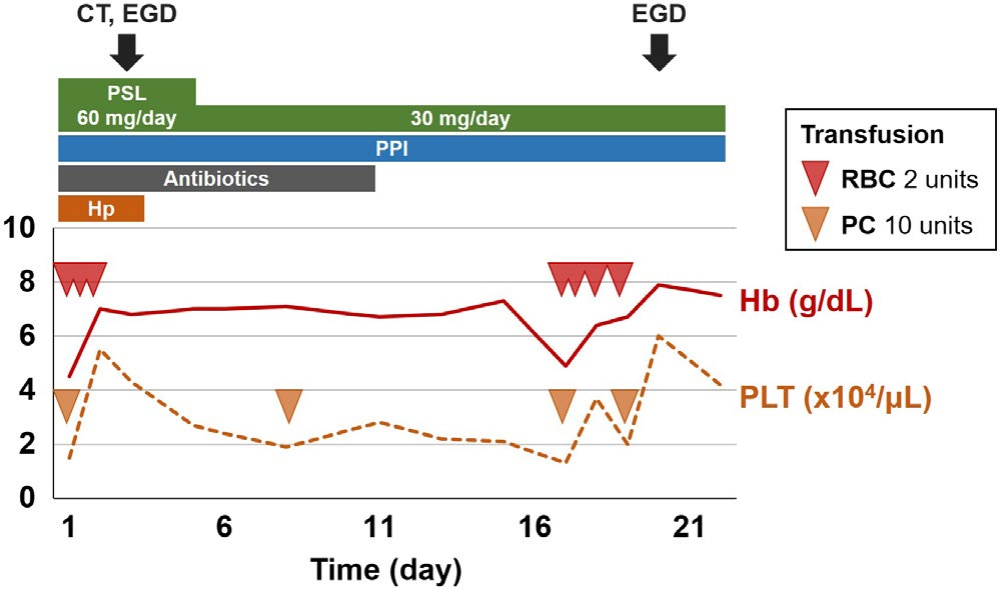
Clinical course. CT, computed tomography; EGD, esophagogastroduodenoscopy; Hb, hemoglobin; Hp, haptoglobin; PC, platelet concentrate; PLT, platelet count; PPI, proton‐pump inhibitor; PSL, prednisolone; RBCs, red blood cells

**Figure 2:**
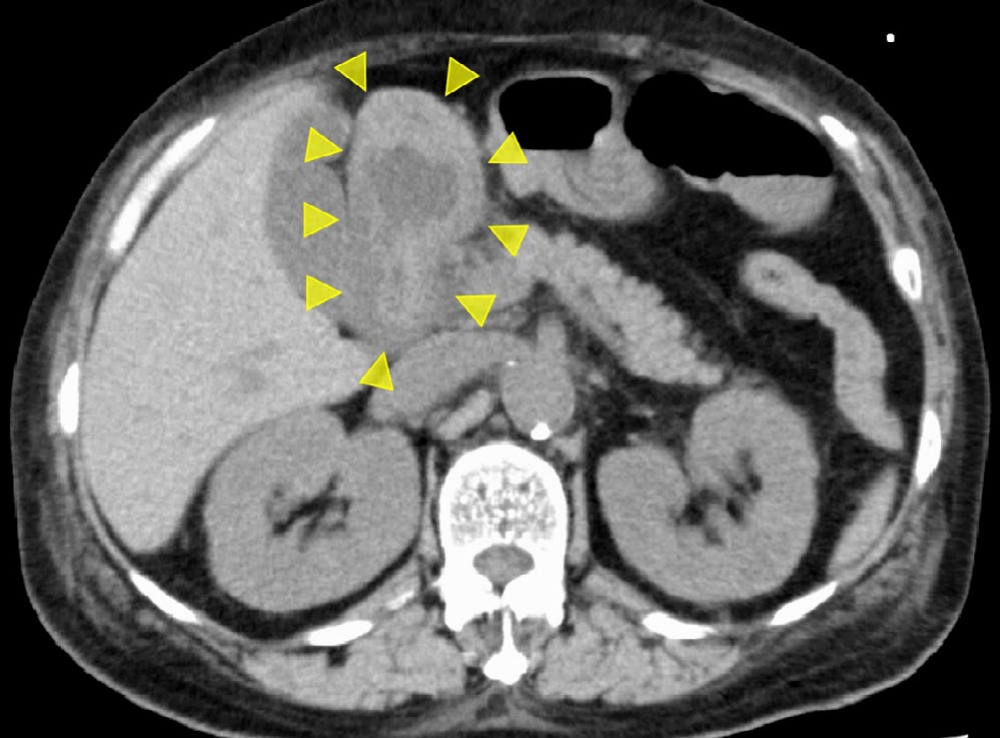
CT image. The duodenal wall is markedly thickened (arrowheads)

**Figure 3:**
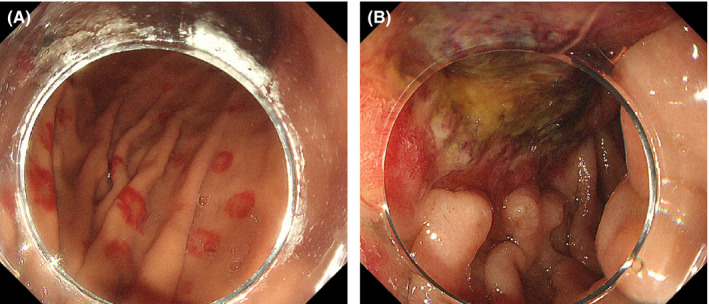
Endoscopic images at presentation. (A) Target‐like patchy redness in the body of the stomach. (B) A large punched‐out ulcer at the superior duodenal angle

She was admitted, and we started intravenous administration of prednisolone, lansoprazole, and ceftriaxone (Figure 1). Blood products, including haptoglobin, were also given. Two days later, hematologic and inflammatory markers improved; however, she developed upper abdominal pain and vomiting. Computed tomography (CT) revealed marked thickening of the duodenal wall (Figure 2). Esophagogastroduodenoscopy (EGD) revealed patchy redness of the gastric mucosa (Figure 3A) and a large punched‐out ulcer in the duodenum (Figure 3B), with clear demarcation between normal and affected mucosa. Biopsy specimens had no specific findings‐malignant cells, granuloma, nor viral inclusion bodies. Thus, we suspected transient ischemia due to PNH‐related thrombosis had caused the gastroduodenal injury.

**Figure 4:**
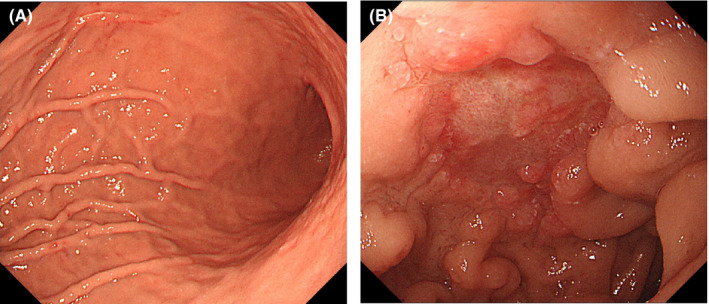
Endoscopic images at follow‐up. (A) Patchy redness in the stomach has disappeared. (B) Duodenal ulcer is in a healing stage

Because there were no signs suggestive of bowel obstruction, perforation, or uncontrolled bleeding, we continued therapy for PNH, with corticosteroids, proton‐pump inhibitor, and diet control. Her symptoms improved in a few days. In a follow‐up EGD performed 3 weeks after admission, the gastric patchy redness had disappeared, and the duodenal ulcer had improved (Figure 4). In laboratory tests, fibrin‐related markers also normalized (Table 1). After vaccination against meningococcus, eculizumab was added to the therapy to achieve better control of the PNH.

## 3 DISCUSSION

To our best knowledge, this is the first report of endoscopic findings of gastric lesions likely caused by PNH‐related thrombosis; patchy areas of gastric redness were the distinctive features. We hope this report will help clinicians recognize and manage the rare but potentially serious gastrointestinal complications of PNH.

Making the diagnosis of PNH‐related thrombosis can be challenging, mainly because of unfamiliarity with this rare complication. In our patient, the cause of the gastroduodenal lesions in our patient was initially unexplained: *H pylori* had been eradicated; she used nonsteroidal anti‐inflammatory drugs (NSAIDs) only occasionally; she did not take antithrombotic drugs; and she had no abnormal laboratory tests or past history or family history suggesting other gastrointestinal diseases, for example, inflammatory bowel diseases, autoimmune vasculitis, or viral infection. In addition, endoscopic biopsy did not show any disease‐specific findings. She had received prednisolone before endoscopic examination for a few days, but steroids were known to be a high risk of peptic ulcer disease when used together with NSAIDs or for more than a month.^7^, ^8^ The single and short‐term use of prednisolone was not likely to be a cause for ulcers in our case.

PNH‐related thrombosis is rarely encountered in Japan. The prevalence of PNH is 6.93 per million population,^9^ and PNH‐related thrombosis is reported in only 4.3% of PNH patients in Japan.^10^ On the other hand, about half of PNH patients in Western countries die from thrombotic complications^11^; this regional difference is unexplained. Nonetheless, thromboembolic complications are a significant cause of death in PNH. Hemolysis and elevation of D‐dimer levels, noted in our patient, are known as the signs of thrombotic complications.^4^, ^12^ In addition, in PNH‐related thrombosis, intraabdominal veins are most often involved.^13^ Thus, thrombotic gastrointestinal injury should be suspected in patients with PNH presenting with such laboratory findings and gastrointestinal symptoms.

Clinical manifestations and endoscopic findings of PNH‐related upper gastrointestinal lesions are not well known. As far as we know, only six case reports have been published^5^, ^14^, ^15^, ^16^, ^17^, ^18^; their salient findings are summarized in Table 2. According to those reports, any portion of the duodenum was affected, the small bowel was frequently involved simultaneously, and surgical treatment was sometimes required. Pathologically, intravenous thrombosis, vascular proliferation, and papillary endothelial hyperplasia within the duodenal submucosa have been reported^14^, ^15^, ^16^, ^17^; a characteristic CT finding is edematous thickening of the duodenal wall.^5^, ^15^, ^16^, ^17^ Among EGD findings, various degrees of erosion and/or ulceration with clear demarcation have been described.^5^, ^14^, ^15^, ^16^, ^17^ The patchy areas of gastric redness present in our patient have not been reported so far. It seems difficult to make a diagnosis of PNH by endoscopic biopsy; nonetheless, gastrointestinal biopsy may be beneficial in differentiating PNH from IgA vasculitis, which can present with both gastric patchy redness and duodenal ulcer.^19^, ^20^

**Table 2:** Summary of previous reports of upper gastrointestinal complications of PNH

Author	Age/sex	Affected site	Small bowel involvement	CT findings	Endoscopic findings	Treatment
Dunphy CH	17/M	3rd, 4th portion of duodenum	Present	NA	Ulcerating, necrotic, hemorrhagic, restricting mucosal lesions	Surgical
Zapata R	27/F	2nd, 3rd portion of duodenum	Present	Thickening of duodenal wall	Diffuse mucosal edema, multiple erosions Sharp demarcation line	Supportive care, LMWH
Adams T	45/F	2nd portion of duodenum	Present	Thickening of duodenal wall	Mucosal edema, diffuse erythema, and ulcerations	Supportive care, heparin
Quentin V	44/F	4th portion of duodenum	Present	Mucosal edema	Patchy congestive mucosa, ulcerations	Surgical
Torres J	33/F	1st, 2nd portion of duodenum	Absent	Thickening of duodenal wall	Bluish mucosal edema, multiple erosions, and ulcerations Clear demarcation	Supportive care, LMWH
Tezcaner T	49/M	4th portion of duodenum	Absent	Bowel obstruction at distal duodenum	NA	Surgical

Abbreviations: CT, computed tomography; F, female; LMWH, low‐molecular‐weight heparin; M, male; NA, not available.

Treatment with corticosteroids, proton‐pump inhibitors, and diet control was successful in our patient. Various treatments have been reported for PNH‐related thrombosis.^2^ In cases of bowel obstruction or perforation, surgical treatment has been needed^17^, ^18^; in cases without such complication, nonoperative therapy was appropriate.^21^ In patients with life‐threatening thrombosis, anticoagulation therapy is necessary. In Japan, however, PNH patients die from bleeding events more often than from thrombotic complication.^10^ Thus, we avoided anticoagulation therapy. Eculizumab is another treatment option^2^, ^22^; however, at least two weeks after vaccination against *Neisseria meningitides* are required before initiation of eculizumab. We used corticosteroids, expecting a rapid suppression of hemolysis,^23^ but its effect on thrombosis was unclear. Fortunately, corticosteroid therapy together with proton‐pump inhibitor and diet control was adequate in our case. In the absence of definitive treatment, early and accurate diagnosis of thrombotic complications seems important so the thrombotic manifestations can be treated and surgical intervention avoided.

In conclusion, in patients with PNH who have abdominal symptoms, gastrointestinal injury caused by PNH‐related thrombosis should be suspected. In addition to laboratory data suggesting thrombosis, recognition of gastric patchy redness at EGD may help reach the diagnosis of this potentially serious complication of PNH.

## CONFLICT OF INTEREST

The authors declare no conflicts of interest associated with this manuscript.

## AUTHOR CONTRIBUTIONS

MU, KO, and RT: involved in the patient's care. MU and YS: contributed to the conception of the work. MU: drafted the manuscript. YS, KO, RT, HY, and MM: revised the manuscript. All authors: discussed the clinical case, interpreted the findings, and contributed to the final manuscript.

## ETHICAL APPROVAL

Informed consent was obtained from the patient for publication of this case report and accompanying images.

## Data Availability

Nondigital data available.
